# Unusual cardiac outcomes of bacteremia due to *Staphylococcus aureus* infection: a case report

**DOI:** 10.1186/1752-1947-6-326

**Published:** 2012-09-26

**Authors:** Paula Lage Pasqualucci, Vera Demarchi Aiello, Liliane Kopel

**Affiliations:** 1 ABC Medical School, Santo André, SP, Av. Príncipe de Gales, 821, Santo André, SP, 09060-650, Brazil; 2 Laboratory of Pathology, Heart Institute (InCor), Hospital das Clínicas da Faculdade de Medicina da Universidade de São Paulo, Av. Dr. Eneas de Carvalho Aguiar, 44, São Paulo, SP, 05403-900, Brazil; 3 Department of Cardiology, Intensive Care Unit, Heart Institute (InCor), Hospital das Clínicas da Faculdade de Medicina da Universidade de São Paulo, Av. Dr. Eneas de Carvalho Aguiar, 44, São Paulo, SP, 05403-900, Brazil

**Keywords:** Hemodialysis, Sepsis, *Staphylococcus aureus*, Bacterial coronary arteritis, Myocardial abscesses, Papillary muscle rupture

## Abstract

**Introduction:**

Patients on hemodialysis, particularly those with temporary non-tunneled dialysis access, constitute a high-risk population for bloodstream infections associated with significant morbidity and mortality rates. This population also has a high prevalence of cardiovascular diseases with poor prognosis.

**Case presentation:**

We report the case of a 62-year-old Caucasian man with end-stage renal disease undergoing hemodialysis, who presented with an eventually lethal case of staphylococcal septicemia with an unusual involvement of the heart, including bacterial coronary arteritis, myocardial abscesses and papillary muscle infarction and rupture, along with complications involving other organs.

**Conclusions:**

It is important to try to minimize invasive procedures in patients who are hemodialysis dependent. The strict control of heart function is indicated considering the large spectrum of unusual cardiac complications.

## Introduction

Patients on hemodialysis, particularly those with temporary non-tunneled dialysis access, constitute a high-risk population for bloodstream infections associated with significant morbidity and mortality rates. This population also has a high prevalence of cardiovascular diseases with poor prognosis. We describe the case of a patient with end-stage renal disease undergoing hemodialysis, who presented with an ultimately lethal case of staphylococcal septicemia with unusual involvement of the heart, including bacterial coronary arteritis, myocardial abscesses and papillary muscle rupture, along with complications involving other organs.

## Case presentation

A 62-year-old Caucasian man with end-stage renal disease presented to our hospital because of a four-day history of fever, diarrhea and the need for emergency dialysis due to hyperkalemia. Our patient had been treated with hemodialysis for one year. Over the last month, a temporary non-tunneled catheter had been used for vascular access because of arteriovenous graft thrombosis. However, he had missed the two last dialysis sessions. His prior medical history included diabetes mellitus type 2 and systemic arterial hypertension. During the hemodialysis procedure, our patient experienced shock and respiratory insufficiency with a requirement for endotracheal intubation and mechanical ventilation. An electrocardiogram revealed ST-segment depression in leads V4 to V6, suggesting an episode of acute myocardial ischemia. An emergency coronary arteriography was performed and showed severe stenosis of the proximal circumflex and anterior inter-ventricular coronary branches. Percutaneous coronary intervention with successful stent implantation was performed in both arteries. Because of our patient’s poor hemodynamic condition, despite the use of vasopressor and inotropic drugs, an intra-aortic balloon counterpulsation was placed and our patient was admitted to our intensive care unit (ICU). A rise in cardiac troponin and creatine kinase (CK)-MB levels confirmed the diagnosis of myocardial infarction.

Broad-spectrum intravenous antibiotics were initiated because of our patient’s previous history of fever and diarrhea. Peripheral blood cultures obtained at admission tested positive for methicillin-resistant *Staphylococcus aureus* (MRSA). Culture of the removed dialysis catheter tip also grew MRSA.

On the fifth day of our patient’s ICU stay, echocardiography showed relevant pericardial effusion with impaired right ventricular filling. Pericardial drainage was performed and 500mL of purulent fluid drained. Cultures again tested positive for MRSA*.* A transesophageal echocardiogram revealed no valvular vegetation.

On the seventh day after our patient’s admission, ocular opacity was observed. An aqueous humor sample was collected and a culture grown tested positive for MRSA, which allowed a diagnosis of endogenous bacterial endophthalmitis to be made. Neurological clinical examination revealed nuchal rigidity. A lumbar puncture was performed and cerebrospinal fluid culture grew MRSA, confirming the diagnosis of bacterial meningitis. Septic arthritis of the right knee was diagnosed based on a positive synovial fluid culture for MRSA. A computed tomography (CT) scan of the thorax showed multiple cavity nodules in both lungs, and sphenoid sinus tomography showed sphenoid sinusitis, probably of staphylococcal etiology.

On our patient’s 14th day in the ICU, a mitral regurgitation murmur was diagnosed. A second transesophageal echocardiography procedure showed a mobile image attached to the mitral valve leaflet with the appearance of vegetation, and an image compatible with ruptured anterior mitral chordae tendineae, resulting in severe mitral regurgitation. One day later, our patient experienced a refractory shock followed by cardiac arrest non-responsive to resuscitative efforts, and died.

**Figure 1:**
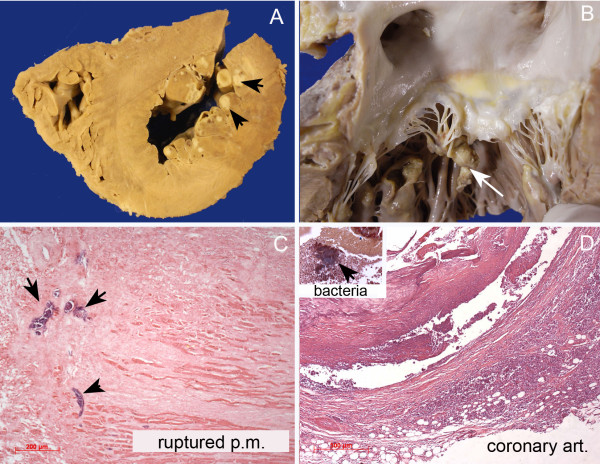
**Gross and histological findings of the heart.** (**A**) This panel shows a short-axis section of the ventricles with small subendocardial abscesses (arrows). In (**B**) the left atrium and the mitral valve were opened. There is complete rupture of the anterior papillary muscle, with the tip still bound to the mitral chordae (white arrow). (**C**) This panel shows a photomicrography image of the necrotic papillary muscle (p.m.). The arrows point to groups of cocci. In (**D**), part of the wall of the anterior descending coronary arterial (coronary art.) branch shows a dense neutrophil exudate. The insert shows a group of bacteria found among the inflammatory cells (Brown-Hopps stain).

An autopsy was performed and our patient’s heart was observed to have moderate enlargement. The pericardial surface was covered with greenish granular exudate and loose adhesions were present between the visceral and parietal layers of the pericardium. Concentric hypertrophy with mild dilatation of the left ventricle was observed. The subendocardial layer of the anterior, lateral and inferior walls of the left ventricle showed many yellowish areas with softened granular centers (Figure 1A). Complete rupture of the anterior papillary muscle was also found (Figure 1B), but the mitral valve leaflets did not present vegetation. The aortic, tricuspid and pulmonary valves were normal. Microscopic examination revealed acute purulent pericarditis and multiple subendocardial abscesses with Gram-positive cocci colonies within areas of healing necrotic myocardium, including the ruptured papillary muscle (Figure 1C). Histological evaluation of the coronary arteries showed an acute inflammatory process in the wall of the circumflex and anterior inter-ventricular branches, characterized by neutrophil infiltration and the presence of Gram-positive cocci (Figure 1D). Abscesses were also found in the lungs and spleen. Pulmonary edema was present.

## Discussion

Our patient presented with septicemia caused by *S. aureus*, with a clinical and histopathological diagnosis of systemic infection. Clinical findings of arthritis, meningitis and endophthalmitis clearly demonstrated widespread staphylococcal infection with bacteriological diagnoses of *S. aureus* etiology. On autopsy, lung and spleen abscesses were also found. However, the most important findings of our case are the unusual cardiac complications seen on autopsy. Although infectious endocarditis would be the first hypothesis to explain the mitral regurgitation, it was not found, and papillary muscle rupture was the consequence of myocardial infarction due to septic emboli. The coronary arterial walls also showed infectious complication characterized by acute arteritis due to bacterial dissemination.

End-stage renal disease is accompanied by disturbances of the immune system caused by several mechanisms [1]. The presence of diabetes mellitus confers additional risk for death related to septicemia. Higher rates of bloodstream infection have also been demonstrated in patients with temporary dialysis catheters compared to those with arteriovenous grafts [2,3]. All the evidence supports the hypothesis that our patient’s dialysis catheter was the origin of his staphylococcal septicemia. Infective endocarditis has a high incidence in patients on hemodialysis with an increased proportion of *S. aureus* etiology, however coronary arteritis due to infectious agents is rare [4].

An autopsy study of our patient demonstrated infectious coronary arteritis. The infection route was probably due to hematogeneous dissemination of bacteria, but we cannot rule out the possible role of coronary percutaneous procedure as participating in the infectious spread. The degree of atherosclerotic involvement of the coronary arteries could not be evaluated due to the process of acute arteritis.

Besides the septicemia, the final cause of death in our patient was certainly related to the infarction and rupture of the papillary muscle with consequent sudden onset of severe mitral regurgitation, which led to acute heart failure [5].

## Conclusions

In order to decrease the rate of bacteremia in patients who are hemodialysis dependent, we should try to minimize invasive procedures and the use of temporary non-tunneled catheters for dialysis, and maintain strict prophylactic measures against catheter-related infections. In addition, the strict control of heart function is indicated considering the large spectrum of unusual cardiac complications.

## Consent

Written informed consent was obtained from the patient’s next-of-kin for publication of this manuscript and any accompanying images. A copy of the written consent is available for review by the Editor-in-Chief of this journal.

## Competing interests

The authors declare that they have no competing interests.

## Authors’ contributions

PLP made a substantial contribution to the conception of the paper and acquisition of data, and was involved in drafting the manuscript. VDA made a substantial contribution to the acquisition of autopsy data and was involved in drafting the manuscript. LK made a substantial contribution as assistant physician to our patient, to the acquisition of data, and was involved in drafting the manuscript. All authors read and approved the final manuscript.

## References

[B1] Jaber BL Bacterial infections in hemodialysis patients: pathogenesis and prevention Kidney Int 2005 67 2508 2519 10.1111/j.1523-1755.2005.00364.x 15882306

[B2] Lok CE Mokrzycki MH Prevention and management of catheter-related infection in hemodialysis patients Kidney Int 2011 79 587 598 10.1038/ki.2010.471 21178979

[B3] Patel PR Kallen AJ Arduino MJ Epidemiology, surveillance, and prevention of bloodstream infections in hemodialysis patients Am J Kidney Dis 2010 56 566 577 10.1053/j.ajkd.2010.02.352 20554361

[B4] Dishop MK Yoneda K Staphylococcal coronary arteritis as a complication of septicemia Arch Pathol Lab Med 1999 123 332 334 10320146 10.5858/1999-123-0332-SCAAAC

[B5] Aiello VD Mansur AJ Favaratto D Rupture of posteromedial papillary muscle as a mechanism of death in dilated cardiomyopathy Int J Cardiol 1996 54 73 75 10.1016/0167-5273(96)02559-4 8792188

